# Tuberculous abdominal cocoon mimicking peritoneal carcinomatosis

**DOI:** 10.1186/s13756-019-0562-y

**Published:** 2019-06-19

**Authors:** Zhi-Xin Meng, Yan Liu, Rui Wu, Kai Shi, Tao Li

**Affiliations:** 1 Department of General Surgery, Qilu Hospital, Shandong University, 107 West Wen Hua Road, Jinan, 250012 China; 2 Inpatient department, Qilu Hospital, Shandong University, Jinan, 250012 China

**Keywords:** Abdominal cocoon, Tuberculosis, Intestinal obstruction

## Abstract

**Background:**

Tuberculous abdominal cocoon is an uncommon manifestation of abdominal tuberculosis. As a rare clinical entity, it is often encountered unexpectedly in patients with small intestinal obstruction. Here we presented a rare case of tuberculous abdominal cocoon which was suspected to be peritoneal carcinomatosis and was finally diagnosed by laparoscopy.

**Case presentation:**

A 47-year-old man developed small intestinal obstruction and massive ascites that did not resolve with conservative management. Surgical exploration revealed a fibrous sheath covering the small-bowel, and pathologic assessment of biopsies confirmed intra-abdominal tuberculous infection. After antituberculosis therapy, the ascites has greatly diminished and the patient was functioning normally.

**Conclusion:**

Preoperative diagnosis of tuberculous abdominal cocoon is a true challenge. Early diagnostic peritoneal biopsy should be recommended and surgery is usually unnecessary if definitive diagnosis can be made.

## Backgrounds

Abdominal cocoon, also known as sclerosing peritonitis or sclerosing encapsulating peritonitis, is a rare cause of small intestinal obstruction and is characterized by total or partial encasement of the small bowel by a thick, fibrous, cocoon-like membrane [1]. Abdominal cocoon can be classified as primary (idiopathic) or secondary according to the etiology [2, 3]. Tuberculosis as an etiology of secondary abdominal cocoon has been increasingly recognized particularly in the developing world where extrapulmonary tuberculosis has an increasing epidemiological trend. Awareness of the clinical features of tuberculous abdominal cocoon is crucial for improving diagnostic accuracy and survival.

## Case report

**Fig. 1:**
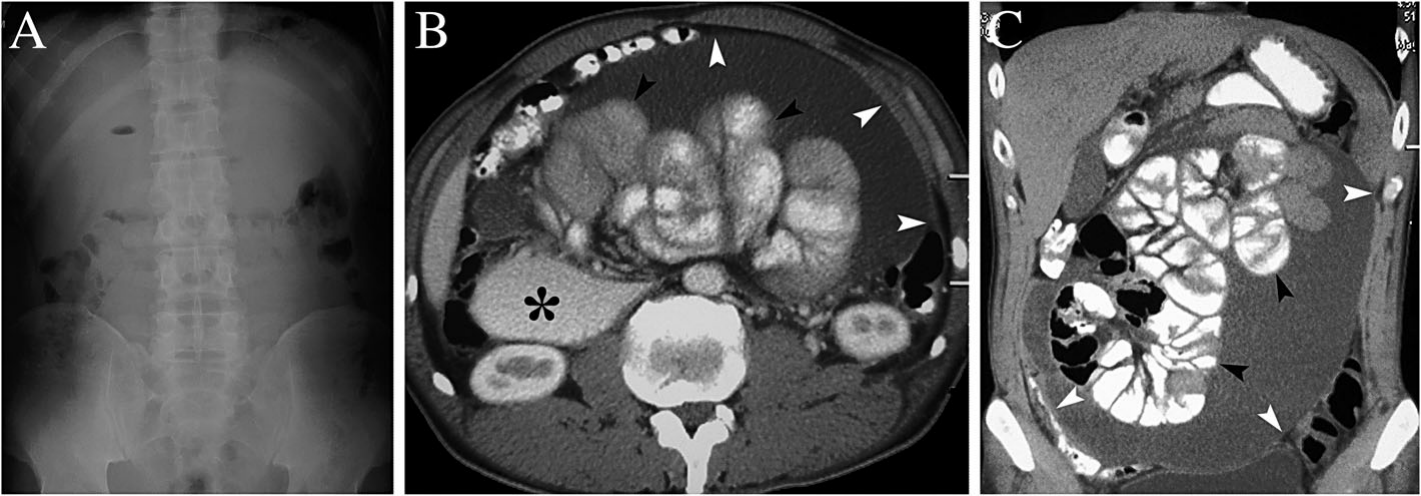
**a**: Plain upright abdominal X-ray showed some air in the colon without presence of multiple air fluid levels in the loops or free gas under the diaphragm; **b**, **c**: Contrast-enhanced computed tomogram scan revealed dilatation of the duodenum loops (*) and congregated small gut loops (black arrowhead) trapped in the massive ascites surrounded by a membrane (white arrowhead)

A 47-year-old man was referred to our hospital with a 3-month history of abdominal distension, intermittent abdominal pain and nausea. Despite weight loss of 4 kg, the patient had no symptom of fever, chronic cough or night sweats. He had no history of abdominal surgery, liver cirrhosis or chronic hepatitis virus infection, but suffered from tuberculous pleurisy about 20 years ago. Physical examination revealed gross abdominal distension without tender and ascites of unknown aetiology. Laboratory blood analyses including serum tumor markers, erythrocyte sedimentationrate (ESR), adenosine deaminase activity (ADA) and anti-tuberculosis antibody (TB-Ab) were all within normal limits, and tuberculin skin test was negative. The results of antibody testing for HIV were negative. Chest radiograph did not demonstrate features suggestive of pulmonary tuberculosis. Plain upright abdominal X-ray showed some air in the colon without presence of air fluid levels in the loops or free gas under the diaphragm (Fig. 1a). Contrast-enhanced computed tomogram (CT) of the abdomen (Fig. 1b, c) revealed dilatation of the duodenum loops (*) and congregated small gut loops (black arrowhead) trapped in the massive ascites surrounded by a membrane (white arrowhead). Gastroscopy revealed no signs of malignancy. A peritoneal tap was performed twice and yielded blood stained ascitic fluid but no malignant cells or acid-fast bacilli. The ascities was exudates in nature and ascities ADA was within normal ranges. The patient refused laparoscopy examination and was discharged without a definitive diagnosis and further treatment.

**Fig. 2:**
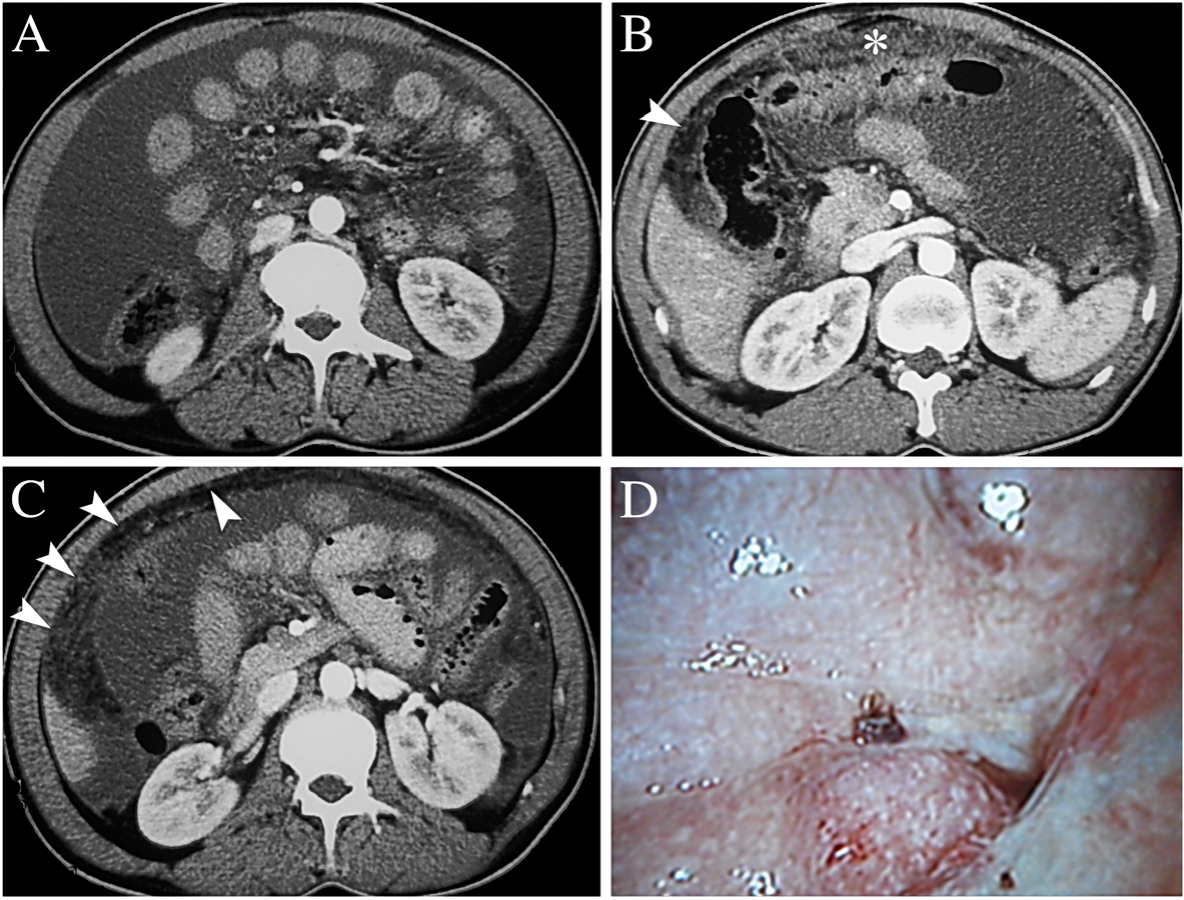
**a**: CT scan showed the presence of well-encapsulated fluid collection and central accumulation of the small intestine; **b**, **c**: CT scan revealed smudged appearance of the greater omentum (*), as well as multiple small nodules and sheetlike lesions on the parietal peritoneum (arrowhead); **d**: Laparoscopy demonstrated that the entire small bowel was encapsulated in a dense, white, fibrous, cocoon-like membrane associated with a large amount of brownish ascites. Multitudinous miliary nodules or ubercles were seen on the parietal peritoneum

Two months later, the patient was presented to our hospital again with persistent symptoms. Despite abdominal distension, nausea and vomiting, he also complained of increasing fatigue, emaciation, and 10-kg weight loss for the recent two months. Laboratory blood analyses revealed that serum ADA was 21 U/L (normal range 4–18 U/L), ESR was 28 mm/h (normal range 0–15 mm/h), but TB-Ab was negative. Ascitic fluid obtained by paracentesis was still negative for bacteria, acid-fast bacilli and malignant cells, but ascities ADA was significantly elevated (41 U/L). Serum CA-125 was also significantly elevated (97.39 U/ml, normal range < 35 U/L). In addition, despite the presence of well-encapsulated fluid collection and central accumulation of the small intestine (Fig. 2a), CT scan also revealed smudged appearance of the greater omentum (Fig. 2b,*), as well as multiple small nodules and sheetlike lesions on the parietal peritoneum (Fig. 2. s, arrowhead), which the radiologists considered to be peritoneal carcinomatosis (PC). Mesenteric lymphadenopathy was not observed on CT scan. The presumptive diagnosis was PC or tuberculous peritonitis in our patient, however, other differential diagnosis, such as peritoneal mesothelioma, could not be completely ruled out.

Diagnostic laparoscopy was performed in our patient, and the entire small bowel was found to be encapsulated in a dense, white, fibrous, cocoon-like membrane filled with a large amount of brownish ascites (Fig. 2d). Multitudinous miliary nodules or tubercles were seen on the parietal peritoneum and greater omentum. Biopsy of the nodules and plaques revealed caseous granulomatous inflammation and the specimen were stained positive for acid-fast bacilli. Histopathology of the cocoon wall revealed fibrocollagenic tissue with granulomatous inflammation, and culture of the biopsied specimen grew *Mycobacterium tuberculosis* 3 weeks later. A diagnosis of tuberculous abdominal cocoon was made. Complete excision of the thick membrane and lysis of adhesions were tried but failed due to the extreme difficulty in separating the fibrotic tissue from the abdominal wall and small bowel. Since there were no perforated, ischemic or dilated bowel loops, the operation was completed after obtaining samples for diagnostic biopsy.

**Fig. 3:**
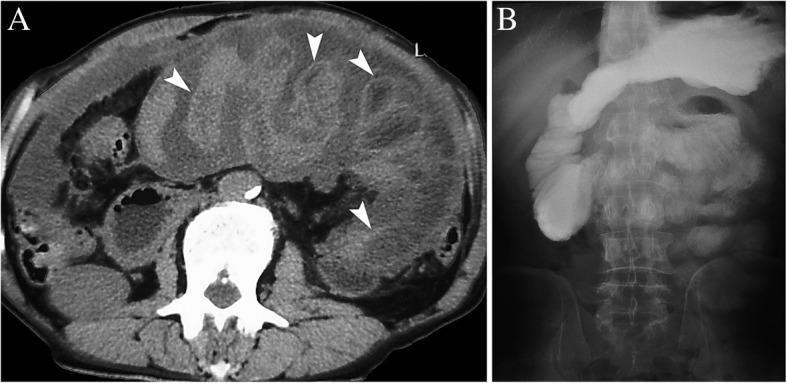
**a**: CT scan showed the small bowel wall thickening and membrane covering the small bowels (arrowhead), and the ascites has greatly diminished; **b**: Gastrografin meal follow through study revealed adherent small bowel loops with delayed transit time

After operation, the patient had recurrent episodes of partial bowel obstruction that was resolved with conservative management. The patient was given rifampicin, isoniazid, ethambutol, and pyrazinamide for 2 months, followed by rifampicin and isoniazid for 4 months. By the end of the 2 month of anti-tuberculous therapy, serum ADA, ESR and CA 125 levels have returned to normal. Though a repeat CT scan of the abdomen and pelvis continued to show the small bowel wall thickening and membrane covering the small bowels (Fig. 3a, arrowhead), and gastrografin meal follow through study revealed adherent small bowel loops with delayed transit time (Fig. 3b), however, the ascites has greatly diminished and the patient was functioning normally. No further surgical intervention was planned for him, and he was followed up symptom-free for 6 years.

## Discussion

With the introduction of effective antituberculosis chemotherapy and improvements in the socioeconomic environment, the incidence of tuberculosis has been decreasing [4]. However, extrapulmonary tuberculosis has emerged as a devastating disease during the last decade with a high morbidity and mortality both in developing and developed countries due to HIV epidemic, immigration spreads and drug resistance [5, 6]. Peritoneal tuberculosis is an uncommon site of extrapulmonary tuberculosis and is usually secondary to hematogenous spread from pulmonary lesions, but may also occur due to spread from subjacent bowel involvement, lymph nodes or from fallopian tubes in females [4].

Because peritoneal tuberculosis may have an insidious onset and nonspecific clinical presentation, early diagnosis is often difficult to make. A negative smear for acid-fast bacillus, a lack of granulomas on histopathology, and failure to culture *Mycobacterium tuberculosis* do not exclude the diagnosis. Direct microscopic smear detection of acid-fast bacilli in the ascitic fluid is insensitive, with sensitivity ranging from 0 to 6% [7]. Ascites ADA has been reported to have a sensitivity of 100% and specificity of 96% in discriminating tuberculosis peritonitis from other causes of ascites [8], but CA-125 levels should be cautiously interpreted in differentiating between peritoneal tuberculosis and PC, as both of them have elevated serum and ascites CA-125 levels [9]. Peritoneal biopsy provides a diagnostic accuracy of 85–95% for tuberculous peritonitis, and is considered to be a safe and effective method of obtaining an early diagnosis [7]. As delayed initiation of therapy was associated with higher mortality rates of tuberculous peritonitis, peritoneal biopsy should be considered at an early stage to reduce the time from symptom onset to initiation of treatment [4, 7].

The most common symptoms of peritoneal tuberculosis were abdominal distension, weight loss, fever, abdominal pain and abdominal mass, obstruction caused by tuberculous abdominal cocoon is extremely rare. Encasement of varying lengths of small bowel in a fibrocollagenic cocoon-like sac is a pathognomonic feature of abdominal cocoon [10]. Though characteristic CT findings, such as central accumulation of the small intestine encased by a dense membrane with a contrast-free periphery (cauliflower sign), ascites, thickening of the small bowel wall, mural or peritoneal calcifications, localized fluid collection and lymphadenopathy contribute to the diagnosis of tuberculous abdominal cocoon, definitive diagnosis is often made by a combination of the medical history, a high clinical index of suspicion, various biochemical parameters, and examination of peritoneal biopsy specimens [2, 3, 11, 12].

Undiagnosed and untreated tuberculous peritonitis can result in a mortality rate of 50–60% [7], but the disease is usually curable when properly treated. Antituberculosis therapy (ATT) without surgery can lead to the recovery of most tuberculous abdominal cocoon patients [13, 14], and surgery is usually unnecessary if definitive diagnosis can be made preoperatively. In an earlier report on tuberculous intestinal obstruction related to strictures, clinical improvement occurred in 91% patients with ATT while complete radiological resolution was noted in 70% patients, suggesting the role of ATT in management of tubercular strictures [15]. Though ATT is empirically administered to some patients with intestinal obstruction in tuberculosis epidemic areas, the medical history of the patient, ADA level in ascitic fluid, culture of sputum and ascitic fluid, and ESR should be evaluated to avoid erroneous administration.

Despite ATT, conservative treatment such as bowel rest, nasogastric decompression, and nutritional support also contribute to ameliorating abdominal symptoms [16]. Tamoxifen, steroids, colchicine, azathioprine or mycophenolate mofetil are sometimes administrated, to suppress the inflammatory process, inhibit fibroblastic production, collagen synthesis and maturation, and eliminate the thickened membrane [16]. For those presents a diagnostic dilemma and laparoscopy is performed, only biopsies should be taken, dissecting the membranes, separation of adhesions, and bowel resection is usually unnecessary for tuberculous abdominal cocoon, unless there are urgent complications requiring such intervention. Severe adhesions between the bowel wall and membrane make the separation very hazardous, with the potential risk of perforation, development of new adhesions and postoperative intestinal obstruction.

## Conclusion

In summary, though various biochemical parameters and characteristic radiological findings of CT scan play a major role in establishing the diagnosis of tuberculous abdominal cocoon, preoperative diagnosis is a true challenge. The first and most important step towards diagnosis of tuberculous abdominal cocoon is awareness and clinical suspicion proportional to experience and knowledge of the disease. Early diagnostic peritoneal biopsy for patients with unexplained ascites should be recommended, especially in localities where tuberculosis is prevalent.

## Data Availability

Not applicable to this article as no datasets were generated or analyzed.

## Abbreviations

ADA: adenosine deaminase activity
ATT: Antituberculosis therapy
ESR: Erythrocyte sedimentationrate
TB-Ab: anti-tuberculosis antibody
